# Epigenetic Regulation Mechanisms of the Cofilin-1 Gene in the Development and Differentiation of Bovine Primary Myoblasts

**DOI:** 10.3390/genes13050723

**Published:** 2022-04-21

**Authors:** Yujia Sun, Yaoyao Ma, Tianqi Zhao, Mingxun Li, Yongjiang Mao, Zhangping Yang

**Affiliations:** 1 Joint International Research Laboratory of Agriculture and Agri-Product Safety, The Ministry of Education of China, Institutes of Agricultural Science and Technology Development, Yangzhou University, Yangzhou 225009, China; ysunshine@yzu.edu.cn (Y.S.); mayao13403894727@163.com (Y.M.); tianqizhao2021@163.com (T.Z.); 2 Key Laboratory of Animal Genetics & Breeding and Molecular Design of Jiangsu Province, Yangzhou University, Yangzhou 225009, China; limingxun@yzu.edu.cn (M.L.); cattle@yzu.edu.cn (Y.M.)

**Keywords:** epigenetic regulation, cofilin-1, cell differentiation, bovine primary myoblasts

## Abstract

As the quality of beef products has received increasing attention, it is essential to explore the underlying transcriptional and epigenetic mechanisms of meat traits. Our project uses Qinchuan cattle as the research subject. First, we examined the spatiotemporal expression pattern of the *CFL1* gene in a panel of fetal bovine, calf, and adult cattle samples. Then, we performed DNA methylation experiments of *CFL1* on myogenesis and muscle maturation using the BSP amplification and COBRA sequencing techniques and found that high DNA methylation levels showed low expression levels. Next, we performed an assay between bta-miR-182 and the *CFL1* gene and demonstrated that miR-182 could promote bovine primary myoblast differentiation by negatively regulated the expression of *CFL1*. Finally, we constructed an adenovirus overexpression and interference vector and found that *CFL1* could suppress the differentiation of bovine primary myoblasts. In summary, our experiment comprehensively analyzes the epigenetic regulation mechanisms of the *CFL1* gene in the development and differentiation of bovine primary myoblasts. This has far-reaching significance for improving the meat production and meat quality of Qinchuan cattle. This can provide reliable data support and a theoretical research basis for the rapid and efficient breeding selection of local yellow cattle and the genetic improvement of meat quality.

## 1. Introduction

Epigenetics has recently evolved from a collection of diverse phenomena to a defined and far-reaching field of study [1]. The growth and development of skeletal muscle is the primary factor in agricultural meat production and meat quality. Therefore, it is important to research the molecular regulation mechanism of meat traits from the angle of genetics and epigenetics [2]. As the quality of beef products has received increasing attention, studies have shown that the structure of muscle composition and the histological characteristics of muscle fibers are closely related to meat quality. Moreover, the cofilin gene is believed to function as an actin assembly regulator in muscle tissue, playing a key role in the function and regeneration of normal muscles. *Cofilin-1* is ubiquitously expressed in most cell types throughout development and adulthood, and it plays an important role in cell migration, proliferation, phagocytosis, and the development of various types of cancers [3].

In mammals, cofilin-1 is also named N-cofilin (non-muscle type cofilin, NM-type *cofilin*, *cofilin-1*, *CFL1*) [4]. The *cofilin-1* gene is located on chromosome 11q13 and is mainly expressed in various non-muscle tissues, especially in the liver and brain [5,6]. Studies have shown that mammalian homologous cofilin-ineffective yeast cells cannot survive autonomously; however, the expression of mammalian *CFL1* can rescue these mutant cells [7,8].

Studies have shown that the expression of cofilin changes with the growth and development of healthy mouse muscle cells and can gradually transition from the initial NM-type *CFL1* to the late M-type (muscle type) *CFL2* [4]. The *CFL1* gene and the expression of the *CFL2* gene can be detected in skeletal muscle during embryonic development, but with the development of muscle growth, the *CFL2* gene gradually replaces the expression of the *CFL1* gene, until the expression of the *CFL1* gene disappears [9]. Therefore, in adult skeletal muscle, the main detection is *CFL2* (wherein 30~50% is phosphorylated) gene expression, and almost no detection of *CFL1* gene expression. However, taking mouse embryos as the research object, ADF-/- knockout embryos have no effect on the survival of mouse embryos and can complete the following embryonic development function normally [10]. However, the CFL1-/- knockout embryo was unable to complete normal embryo development [11]. For brain-specific CFL1-/- knockout mice, the cell cycle and cell migration of the cerebral cortex are affected to different degrees, indicating that *CFL1* plays an important role in regulating the migration of cerebral cortical neurons during the development of mouse embryos [12]. In a study of the mechanism by which *CFL1* regulates the development of cancer, *CFL1* can induce epithelial interstitial transformation in gastric cancer cells through skeletal recombination, and silencing *CFL1* can inhibit tumor metastasis [13].

However, it is not clear how the *CFL1* gene is involved in the growth and development of myoblasts. Therefore, our project takes local Qinchuan (QC) cattle as the research subject. We examined the spatiotemporal expression pattern of the *CFL1* gene and performed DNA methylation experiments of *CFL1* on myogenesis and muscle maturation, and performed an assay between bta-miR-182 and the *CFL1* gene, and studied the function of the *CFL1* gene on the differentiation of bovine primary myoblasts. This work comprehensively analyzes the mechanism of the *CFL1* gene from the point of view of genetics and epigenetics and further studies the molecular genetic regulation network of the *CFL1* gene. This has far-reaching significance to improve the meat production and meat quality of QC cattle and can provide reliable data support and a theoretical research basis for the rapid and efficient breeding of local yellow cattle and the genetic improvement of meat quality.

## 2. Materials and Methods

### 2.1. Ethics Approval and Consent to Participate

All animal experiments were carried out in accordance with the guidelines of the Institutional Administrative Committee and Ethics Committee of Laboratory Animals (license number: SYXK [Su] 2017-0044) and were approved by the Yangzhou University Institutional Animal Care and Use Committee.

### 2.2. Tissue Preparation and Cell Culture

Samples of seven tissues (heart, liver, spleen, lung, kidney, fat, and muscle) from nine individuals (three individuals per stage) were harvested for RNA isolation (Figure S1) within 10 min after slaughter at three key stages of myogenesis and muscle maturation: 90 days at embryo (fetal bovine, FB), 1 month after birth (calf), and 24 months old (adult bovine, AB).

The HEK293 and C2C12 cell lines were provided by our laboratory. Bovine primary myoblasts were isolated and cultured from bovine longissimus muscle as previously described [14]. The cell lines were cultured in high-glucose Dulbecco’s modified Eagle’s medium (DMEM, HyClone, Logan, UT, USA) containing 10% fetal bovine serum (FBS, Gibco, Carlsbad, CA, USA). Bovine primary myoblasts were generated using high-glucose DMEM with 20% FBS (Gibco, Carlsbad, CA, USA). All cells were cultured at 37 °C containing 5% CO_2_ (Figure S2).

### 2.3. Vector Construction

For overexpression of the bovine *CFL1* gene, we designed and synthesized *CFL1* primers containing *Kpn*I and *Hin*dIII (TaKaRa, Dalian, China) restriction sites, and the primer sequences are shown in Table 1 (CFL1-CMV-F and CFL1-CMV-R). The cDNA of the bovine *CFL1* gene was cloned from QC cattle longissimus muscle. The coding sequences of *CFL1* were subcloned into the pAdTrack-CMV plasmid vector to construct the recombinant shuttle vector pAdTrack/CMV-CFL1. Then, this vector was homologously recombined with plasmid pAdEasy-1 to generate an adenoviral plasmid in BJ5183 cells. The adenoviral plasmids linearized by *Pac*I (TaKaRa, Dalian, China) were transfected into HEK293 cells to generate the adenovirus pAdEasy-1/pAdtrack-CMV-CFL1 (CFL1-CMV).

For interference with the bovine *CFL1* gene, the BlockiT shRNA interference system was used. We designed and synthesized two pairs of primers for the *CFL1* gene (shCFL1-1F, shCFL1-1R, shCFL1-2F, shCFL1-2R) and one pair of primers for the negative control (NC, shRNA-NC-F, and shRNA-NC-R). All primer sequences contacting the *Bam*HI and *Hin*dIII (TaKaRa, Dalian, China) restriction sites are shown in Table 1. The oligonucleotides were cloned into the pENTR/CMV-GFP/U6 vector to construct shuttle vectors pENTR/CMV-GFP/U6-shCFL1-1 and pENTR/CMV-GFP/U6-shCFL1-2 and then recombined with the adenovirus backbone vector pAD/PL-DEST to produce recombinant vectors pAD/PL-DEST/CMV-GFP/U6-shCFL-1 (shCFL1-1) and pAD/PL-DEST/CMV-GFP/U6-shCFL-2 (shCFL1-2). After detection of the interference efficiency of shCFL1-1 and shCFL1-2, fifth-generation recombinant adenovirus particles (shCFL1-2) were produced and further amplified by transfecting HEK293 cells. The titer of adenovirus reached 1.58 × 10^9^ PFU/mL, as determined by TCID50 assays. The entire process for adenovirus generation and proliferation was performed as previously described [15]. Myoblasts at approximately 80% confluence were transfected with adenovirus supernatant at a multiplicity of infection (MOI) = 200.

The *CFL1* 3′UTR sequence including the miR-182 binding site was amplified using a forward primer and reverse primer (CFL1-wild-F and CFL1-wild-R shown in Table 2). An 8-base deletion in the miR-182 binding site of the *CFL1* 3′UTR was generated with a pair of mutagenic primers (CFL1-mut-F and CFL1-mut-R shown in Table 2). The two fragments were ligated into the 3′-end of the Renilla gene in the psiCHECK-2 dual-luciferase reporter vector (Promega, Madison, WI, USA) using restriction enzymes *Xho*I and *Not*I (TaKaRa, Dalian, China) and then ligated by T4 DNA ligase (TaKaRa, Dalian, China) to produce the vectors psiCHECK-2-CFL1-wild (CFL1-Luc) and psiCHECK-2-CFL1-mutated (CFL1-del Luc). The miR-182 mimic and inhibitor were obtained from GenePharma (Shanghai, China).

### 2.4. Cell Treatment

To detect the transfection efficiency of the recombinant vector, CFL1-CMV, shCFL1-1 or shCFL1-2 was transfected into HEK293 cells. To investigate myocyte differentiation, the growth medium (GM) was changed to differentiation medium (DM, 2% horse serum (HyClone, Logan, UT, USA) instead of 10% or 20% FBS) after cell confluence reached approximately 80%. The C2C12 cell lines were cultured for 1, 2, 4, 6, and 8 days prior to RNA extraction. Meanwhile, CFL1-CMV, pAdTrack-CMV vector (control, CTRL), shCFL1-2 or siRNA negative control (NC) was transfected into bovine primary myoblasts using Lipofectamine 2000 (Invitrogen, Carlsbad, CA, USA) to confirm the regulatory mechanism of the *CFL1* gene on myoblast differentiation. Cells were harvested for RNA and protein extraction at 1, 3, 5, and 7 days. The miR-182 mimic or inhibitor was also transfected into primary myoblasts using Lipofectamine 2000 (Invitrogen, Carlsbad, CA, USA) to confirm the effect of miR-182 on myoblast differentiation. The cells were cultured in DM for 4 days.

### 2.5. Dual Luciferase Reporter Assay

Cells were cultured in 48-well plates until the cell growth reached approximately 80% confluence. The miR-182 (bta-miR-182, *Bos taurus* miR-182) mimic and CFL1-Luc or CFL1-del Luc were co-transfected into cells by Lipofectamine 2000 (Invitrogen, Carlsbad, CA, USA). The transfection reagent was replaced with fresh GM 4~6 h after transfection. Next, the cells were washed with PBS and harvested using 200 mL passive lysis buffer (PLB) 24 h post-transfection and rocked for 30 min at room temperature. Firefly and Renilla luciferase activities were measured using the Dual-Luciferase Reporter (DLR) Assay System (Promega, Madison, WI, USA). Both reporter activities were quantitated within the same sample of lysate prepared from cells co-transfected with psiCHECK-2 control vector and pRL-SV40 vector (Promega, Madison, WI, USA). The firefly luciferase activities were normalized to the Renilla luciferase activities in each well. Firefly luciferase luminescence was quenched by greater than 5 orders of magnitude.

### 2.6. Quantitative Real-Time PCR (qRT-PCR) and Western Blot

Total RNA was extracted from tissues or cells, and then 500 ng total RNA was converted to cDNA using the PrimeScript RT regent Kit (TaKaRa, Dalian, China). Random primers, oligo (dT) or miRNA-specific stem-loop primers were designed and used for reverse-transcribed cDNA (Table 3). Gene-specific primers for *CFL1* genes were designed based on published mRNA sequences using Primer Premier 5.0 software (PREMIER Biosoft International, Palo Alto, CA, USA). Transcription levels were normalized to the levels of the housekeeping genes *GAPDH* and *U6*. The primers are listed in Table 3.

Total proteins were extracted from cells using RIPA protein lysis buffer containing 1 mM PMSF (Solarbio; Beijing, China), and probed with monoclonal rabbit anti-CFL1 (ab131519; Abcam, Cambridge, UK) and anti-GAPDH (ab9485; Abcam, Cambridge, UK). Anti-immune rabbit IgG-HRP (LK2001; Sungene Biotech, Tianjin, China) was used as secondary antibody, antibody-reacting bands were detected using ECL luminous fluid (Solarbio, Beijing, China).

### 2.7. Bisulfite Sequencing Polymerase Chain Reaction (BSP) and COBRA

Longissimus dorsi DNA was harvested from eight male individuals (four individuals per stage) within 10 min after slaughter at two key stages of myogenesis and muscle maturation: 90-day embryo (fetal bovine, FB) and 24 months old (adult bovine, AB). The BSP primers were designed by the online MethPrimer software [16]. The details of BSP-amplified nucleotide sequences of *CFL1* DMR (differentially methylated region) are shown in Table 4. Three independent amplification experiments were performed for the *CFL1* gene in each sample. We sequenced five clones from each independent amplification and cloning set; hence, there were 15 clones for the *CFL1* DMR in each sample.

The COBRA (Combined bisulfite restriction analysis) technique is a variation of bisulfite sequencing and combines bisulfite conversion-based polymerase chain reaction with restriction digestion. The PCR products hold a natural *Taq*I endonuclease restriction site (T^CGA) when the CpG dinucleotides are methylated; otherwise, the site cannot be digested if one or more CpG dinucleotides within its recognition sequence are unmethylated. Therefore, in the mixed population of resulting PCR fragments, the ratio of band intensity of the digested fraction to the combined intensities of both the digested and undigested fractions reflected the levels of DNA methylation on the restriction sites.

### 2.8. Statistical Analysis

All data are presented as the mean ± SE of *n* = 3 independent experiments, each performed in triplicate. If the one-way ANOVA results were significant, Duncan’s multiple-range test was performed for multiple comparisons, with values of *p* < 0.05 and *p* < 0.01 accepted as statistically significant and highly significant, respectively. They were all analyzed by SPSS software (version 18.0). The relative gene expression levels were determined using the 2^−ΔΔCt^ method [17]. GraphPad Prism V8.0 software was used for generating figures.

The final sequence results of BSP were processed by the online software QUMA [18] or BiQ Analyzer [19]. The DNA methylation levels of the BSP sequencing results are output in circles. The significance test of differences in methylation levels was analyzed by SPSS software (version 18.0, SPSS Inc., Chicago, IL, USA).

## 3. Results

### 3.1. Spatiotemporal Expression Patterns of the CFL1 Gene

To understand *CFL1* function, we first examined the expression pattern of *CFL1* in a panel of fetal bovine, calf, and adult cattle samples using qRT-PCR. We calculated the relative mRNA expression level of the *CFL1* gene in seven tissues in three developmental stages in QC cattle, and the average Ct value of lung tissue was used as a benchmark.

**Figure 1:**
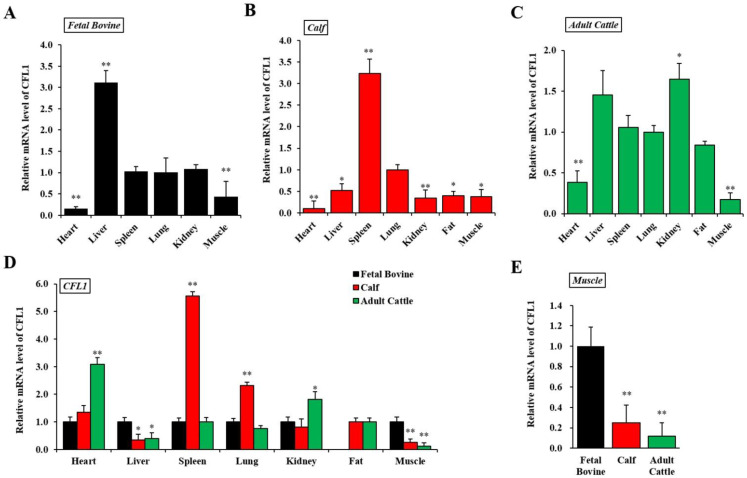
Spatiotemporal expression patterns of the *CFL1* gene in Qinchuan cattle. Relative spatial expression level was analyzed by qRT-PCR in different tissues of three growth and development stages: Qinchuan fetal bovine (**A**), Qinchuan calf (**B**), Qinchuan adult cattle (**C**). The expression levels of lung tissues were considered as 1. Temporal expression patterns of the *CFL1* gene in different tissues of Qinchuan cattle. Relative temporal expression levels in three periods and seven tissues of Qinchuan cattle were investigated using qRT-PCR (**D**,**E**). The expression levels of all tissues in fetal bovine were considered as 1. The mRNA expression level of *CFL1* was normalized to *GAPDH*. Data are means ± SE of *n* = 3 independent experiments, each performed in triplicate. *, *p* < 0.05 and **, *p* < 0.01, two-tailed *t* test.

In fetal bovine, the *CFL1* gene was most highly expressed in liver tissue (3.11 ± 0.29), significantly higher than that of other tissues (*p* < 0.01) and was lowest in the heart (0.15 ± 0.06). The expression level among spleen, lung and kidney tissues were not significantly different (*p* > 0.05) (Figure 1A). In the seven tissues from calves, the *CFL1* gene was most highly expressed in spleen tissue (3.23 ± 0.34), significantly higher than other tissues (*p* < 0.01) and was lowest in the heart (0.10 ± 0.18). The expression levels among liver, kidney, fat, and muscle tissues were not significantly different (*p* > 0.05) (Figure 1B). In the seven adult bovine tissues, the *CFL1* gene was most highly expressed in kidney tissue (1.65 ± 0.19) and was lowest in muscle (0.17 ± 0.08). The expression levels among liver, spleen, lung, and fat tissues were not significantly different (*p* > 0.05) (Figure 1C).

Using the average Ct value of fetal bovine tissues as a benchmark, we further calculated the relative mRNA expression level of the *CFL1* gene in the same tissues in three stages of development in QC cattle, as shown in Figure 1D. In heart tissues, the *CFL1* gene showed the lowest expression level in fetal bovines and the highest in adult bovines.

In liver tissue, the expression level of the *CFL1* gene in the calf and adult bovine stages showed a decreasing trend compared with that of fetal bovine (*p* < 0.05), with the lowest expression level in the calf stage, and the difference of expression level between the calf and adult bovine stage was not significant (*p* > 0.05). In kidney tissue, the expression level of the *CFL1* gene was highest in the adult cattle stage and lowest in the calf stage, and the expression level of the *CFL1* gene showed an overall weak upward trend with growth stage. Compared to the level of expression in embryos and adult bovines, *CFL1* RNA was dramatically upregulated in spleen and lung tissues in calves (*p* < 0.01). In fat samples, because fetal bovines lacked fat deposition, we only detected expression in calves and adult bovines, and the expression levels of the *CFL1* gene were not significantly different between calves and adult bovines (*p* > 0.05). In muscle tissue, the expression level of the *CFL1* gene was the highest in the fetal bovine stage, and the expression level was the lowest in the adult cattle stage, which showed a significant decrease in expression level with age (*p* < 0.01) (Figure 1E).

### 3.2. Study on DNA Methylation Level of CFL1 Gene Promoter

**Figure 2:**
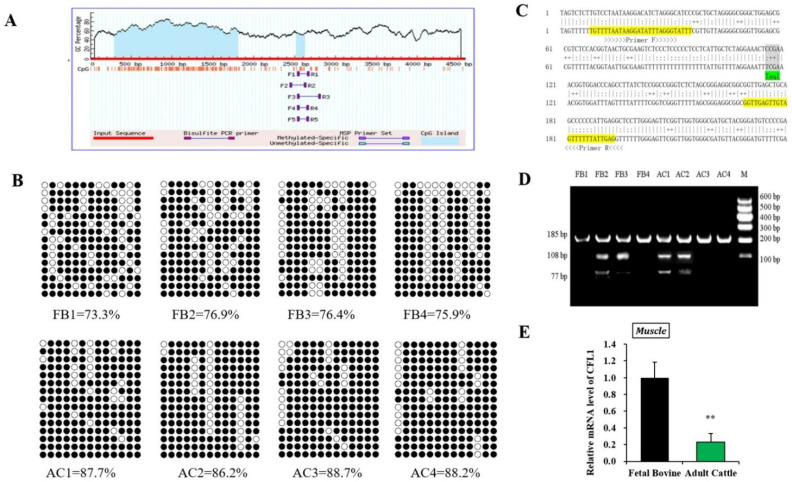
Study on DNA methylation level of *CFL1* gene promoter. Schematic representation of the proximal promoter region (+1 to −4580 base pairs) of the bovine *CFL1* gene (modified output of MethPrimer program) to predict regions of high GC content. The 185 bp CpG islands in that promoter are evident in the 5′ part of the gene (**A**). DNA methylation patterns of muscle tissue in the fetal bovine group and adult bovine group analyzed by BSP. Each line represents one individual bacterial clone, and each circle one single CpG dinucleotide. Open circles show unmethylated CpG’s and black circles methylated CpG’s (**B**). Nucleotide sequences for a 185 bp *CFL1* DMR (total 13 CpG islands) fragment (upper strands) and its bisulfite-converted version (lower strands). Primers sequences are marked with arrows. Squared nucleotides (CpG sites) contain *Taq* I (“TCGA”) (7th CpG site) restriction sites for COBRA analyses (**C**). DNA methylation patterns of muscle tissues in the fetal bovine group (FB, *n* = 4) and adult bovine (AB, *n* = 4) group analyzed by COBRA (**D**). Relative mRNA expression pattern of the *CFL1* gene was analyzed in muscle tissues, the mRNA expression level of *CFL1* was normalized to *GAPDH* (**E**). The data represented the mean ± SE based on three independent experiments (**, *p* < 0.01). M: Marker I.

To confirm the effect of epigenetic modification of the *CFL1* gene on myoblast differentiation, we performed DNA methylation experiments on myogenesis and muscle maturation. The CpG islands located in the *CFL1* DMR were predicted using online software. We found one CpG island in the promoter region of the *CFL1* gene as our research target (Figure 2A). The methylation patterns of the CpG islands were determined using bisulfite-assisted sequencing. The DNA methylation pattern of *CFL1* promoter DMR in the bovine muscle tissues analyzed by BSP is shown in Figure 2B.

Methylation data from BSP sequencing were analyzed by computing the percentage of methylated CpGs of the total number of CpGs using QUMA software. The BSP-amplified sequences of the promoter DMR (185 bp) have 13 CpGs, and we sequenced 15 clones in each muscle sample. Hence, there were 195 CpGs of *CFL1* DMR in each muscle sample, and the percentages of methylated CpGs in the FB group were 73.3%, 76.9%, 76.4% and 75.9%, respectively. The DNA methylation level values in the AC group were 87.7%, 86.2%, 88.7% and 88.2%, respectively. Statistical results showed high DNA methylation levels in the FB group and AC group, whereas the AC group (mean 87.7%) had significantly higher DNA methylation levels than the FB group (mean 75.6%).

To confirm that the BSP sequencing results reflect the overall methylation status, we further performed COBRA analysis using the restriction enzyme *Taq*I (“TCGA”) for *CFL1* promoter DMR (Figure 2C) on the same bisulfite-treated PCR amplification products that were used for the BSP sequencing. The BSP amplified nucleotide sequences, and a cleavage CpG site of the *CFL1* promoter DMR is shown in Figure 2D. At the 7th CpG site, digestion of the 185 bp PCR fragment of *CFL1* DMR with *Taq*I resulted in fragment lengths of 185, 108 and 77 bp bands by COBRA. During sodium bisulfite treatment, unmethylated cytosine residues were converted to “T”, whereas methylated cytosine residues were retained as “C”. The results were consistent with the BSP sequencing results, which confirmed that the BSP sequencing results were reliable. Therefore, in the mixed population of resulting PCR fragments, the fraction that has a cleaved or retained restriction site reflects the percentage DNA methylation in the original genomic DNA.

To further confirm whether the methylation status of the *CFL1* gene affects its level of expression in muscle tissue, we detected the expression level of the *CFL1* gene in the FB group and AC group. The expression level of the *CFL1* gene in the FB group was significantly higher than that in the AC group (*p* < 0.01) (Figure 2E). Combined with the above results, high DNA methylation levels showed low expression levels.

### 3.3. Target Regulation of the CFL1 Gene by microRNA

**Figure 3:**
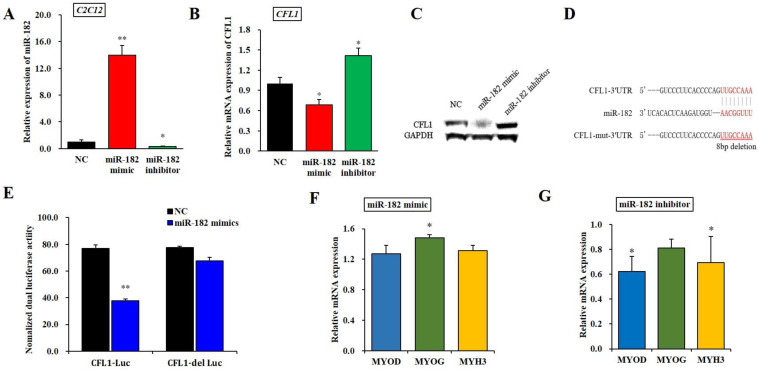
Regulatory relationship between miR-182 and the *CFL1* gene. Expression efficiency of miR-182 was detected by qRT-PCR in C2C12 cells after being transfected with miR-182 mimic and inhibitor for 48 h (**A**). CFL1 mRNA (**B**) and protein (**C**) expression in bovine primary myoblasts were detected by qRT-PCR and western blot after being transfected with miR-182 mimic, miR-182 inhibitor and NC for 48 h. Sequence of miR-182 and its predicted binding site in *CFL1* 3′UTR and mutation 3′UTR (**D**). The miR-182 mimic or NC co-transfected with CFL1-Luc and CFL1-del Luc into bovine primary myoblasts individually, and Renilla luciferase activity was normalized to the firefly luciferase activity (**E**). The expression of myoblast differentiation marker genes *MYOD, MYOG*, and *MYH3* were detected by qRT-PCR after being transfected with miR-182 mimic or inhibitor into C2C12 cells and induced for 4-day by 2% horse serum (**F**,**G**). The expression level of miR-182 was normalized to U6, the mRNA expression levels of *CFL1*, *MYOD*, *MYOG*, and *MYH3* were normalized to GAPDH. Data are means ± SE of *n* = 3 independent experiments, each performed in triplicate. *, *p* < 0.05 and **, *p* < 0.01, two-tailed *t* test. NC, Negative Control; means.

To further determine the effect of epigenetic modification of the *CFL1* gene on myoblast differentiation, we performed a dual-luciferase reporter assay between the miR-182 and *CFL1* genes. First, we predicted the potential miRNAs of the *CFL1* gene using TargetScan7.1, DIANA and miRBase tools and found that miR-182 has a highly conserved binding site in the *CFL1* 3′-UTR. Thus, we inferred that miR-182 might affect the transcription of *CFL1*. To verify this hypothesis, we examined the changes in *CFL1* mRNA expression after transfection with the miR-182 mimic or inhibitor for 48 h. The qRT-PCR assays showed that the expression level of mature miR-182 was significantly higher or lower after transfection of the miR-182 mimic or inhibitor in C2C12 cell lines (*p* < 0.01 or *p* < 0.05) (Figure 3A). The qRT-PCR and western blot analysis indicated that the CFL1 expression level was significantly downregulated or upregulated after transfection of the miR-182 mimic or inhibitor in bovine primary myoblasts (*p* < 0.05) (Figure 3B,C). These results indicated that miR-182 negatively regulated the expression of *CFL1*. The Renilla luciferase activity of bovine primary myoblasts co-transfected with miR-182 mimic and CFL1-Luc was significantly decreased compared to the cells containing only the negative control vector (*p* < 0.01), while luciferase activity was recovered in cells co-transfected with miR-182 mimic and CFL1-del Luc (*p* > 0.05) (Figure 3E). These results demonstrated that miR-182 directly targets *CFL1* by binding to its 3′-UTR.

To determine whether miR-182 affects cell differentiation, C2C12 cells transfected with miR-182 mimic or inhibitor were induced to differentiate for 4 days, and the myoblast differentiation marker genes *MYOD, MYOG*, and *MYH3* were detected by qRT-PCR. We found that the expression of *MYOD* and *MYH3* was significantly downregulated after transfection with the miR-182 mimic (*p* < 0.05), whereas the change in *MYOG* expression level was not significant (*p* > 0.05). Interestingly, the expression of *MYOG* was significantly upregulated after transfection with the miR-182 inhibitor (*p* < 0.05), while the expression levels of *MYOD* and *MYH3* were not significantly changed (*p* > 0.05) (Figure 3F–G). Taken together, these results revealed that miR-182 could promote bovine primary myoblast differentiation.

### 3.4. Effects of the CFL1 Gene on the Differentiation of Myoblasts

**Figure 4:**
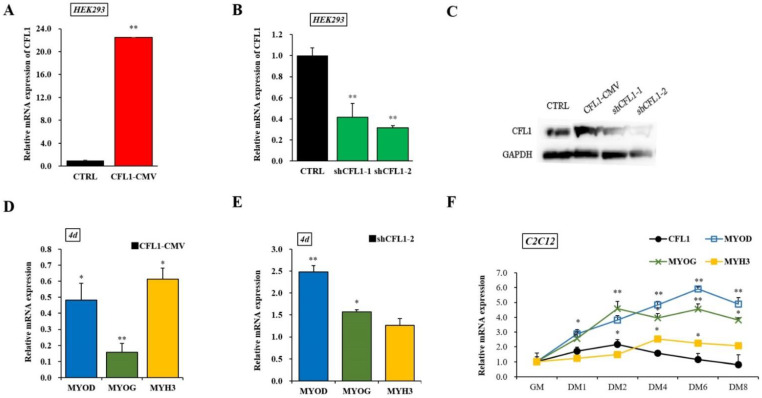
Construction of adenovirus overexpression and interference vectors of the *CFL1* gene. Expression efficiency of CFL1 was detected by qRT-PCR (**A**,**B**) and western blot (**C**) in HEK293 cells after being transfected with adenovirus overexpression (CFL1-CMV) and interference (shCFL1-1 and shCFL1-2) vectors for 48 h. The C2C12 cells were induced to differentiate for 4 days after being transfected with CFL1-CMV or shCFL1-2, and the relative expression of myoblast differentiation marker genes *MYOD*, *MYOG*, and *MYH3* were detected by qRT-PCR (**D**,**E**). Expression trends of the *CFL1* gene and myogenesis factors were detected in C2C12 cells after differentiation had been induced for 1, 2, 4, 6 and 8 days by 2% horse serum (**F**). The mRNA expression level was normalized to *GAPDH*. Data are means ± SE of *n* = 3 independent experiments, each performed in triplicate. *, *p* < 0.05 and **, *p* < 0.01, two-tailed *t* test. CTRL, Control. GM, growth medium. DM, differentiation medium.

To establish the involvement of *CFL1* in myoblast differentiation, we constructed an adenovirus overexpression and interference vector. The qRT-PCR and western blot assays showed that the expression level of CFL1 was significantly increased or decreased after transfection of CFL1-CMV or shCFL1 in HEK293 cells (*p* < 0.01), and shCFL1-2 had a greater interference effect than shCFL1-1 (Figure 4A–C). We transfected CFL1-CMV or shCFL1-2 into C2C12 cells to induce differentiation for 4 days, and the myoblast differentiation marker genes *MYOD*, *MYOG*, and *MYH3* were detected by qRT-PCR. We found that the expression of *MYOD*, *MYOG* and *MYH3* was significantly downregulated after transfection with CFL1-CMV (*p* < 0.05 or *p* < 0.01), and the expression of *MYOG* was significantly decreased compared with *MYOD* and *MYH3*. Interestingly, the expression of *MYOD* was significantly upregulated after transfection with shCFL1-2 (*p* < 0.01), while the expression level of *MYH3* was not significantly changed (*p* > 0.05) (Figure 4D,E). Then, the expression of *CFL1* was quantified in C2C12 cells after differentiation had been induced for 0, 1, 2, 4, 6 and 8 days. We found that *CFL1* was gradually downregulated during myoblast differentiation compared with the level in growth medium (GM), with a slight increase after differentiation Day 2 (DM2). Three marker genes were upregulated during myoblast differentiation. The peak values of *MYOD*, *MYOG* and *MYH3* occurred at DM6, DM2 and DM4 after induction, and expression decreased after DM6 (Figure 4F).

**Figure 5:**
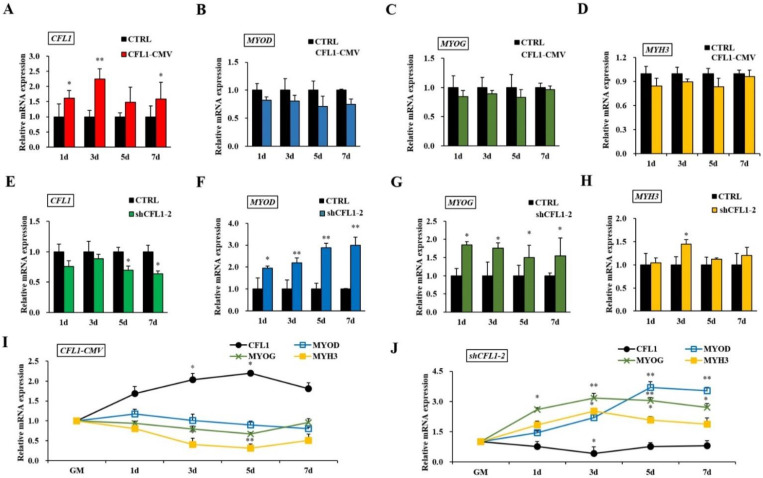
Effects of the *CFL1* gene on the differentiation of bovine primary myoblasts. Expression of *CFL1*, *MYOD*, *MYOG* and *MYH3* were detected by qRT-PCR after being transfected with CFL1-CMV into bovine primary myoblasts and induced for 1, 3, 5, 7 days by 2% horse serum (**A**–**D**). Expression of *CFL1*, *MYOD*, *MYOG* and *MYH3* was detected by qRT-PCR after being transfected with shCFL1-2 into bovine primary myoblasts and induced for 1, 3, 5, 7 days by 2% horse serum (**E**–**H**). Expression trends of *CFL1* gene and myogenesis factors were detected by qRT-PCR after being transfected with CFL1-CMV or shCFL1-2 into bovine primary myoblasts and induced for 1, 3, 5, 7 days by 2% horse serum, the expression levels of four genes in GM were considered as 1 (**I**,**J**). The mRNA expression level was normalized to *GAPDH*. Data are means ± SE of *n* = 3 independent experiments, each performed in triplicate. *, *p* < 0.05 and **, *p* < 0.01, two-tailed *t* test. CTRL, Control. GM, growth medium.

Bovine primary myoblasts are an excellent model system for studying muscle cell differentiation in vitro. After transfection with CFL1-CMV, we found that *CFL1* was significantly upregulated at 1 d, 3 d and 7 d (*p* < 0.05 or *p* < 0.01) (Figure 5A). However, the expression levels of *MYOD*, *MYOG* and *MYH3* were not significantly different in the treatment group (*p* > 0.05) (Figure 5B–D). After transfection with shCFL1-2, CFL1 was significantly downregulated at 5 d and 7 d (*p* < 0.05) (Figure 5E), and *MYH3* was significantly upregulated at 3 d (*p* < 0.05) (Figure 5H). Meanwhile, the expression levels of *MYOD* and *MYOG* were significantly upregulated on each day of myoblast differentiation (*p* < 0.05 or *p* < 0.01) (Figure 5F,G).

**Figure 6:**
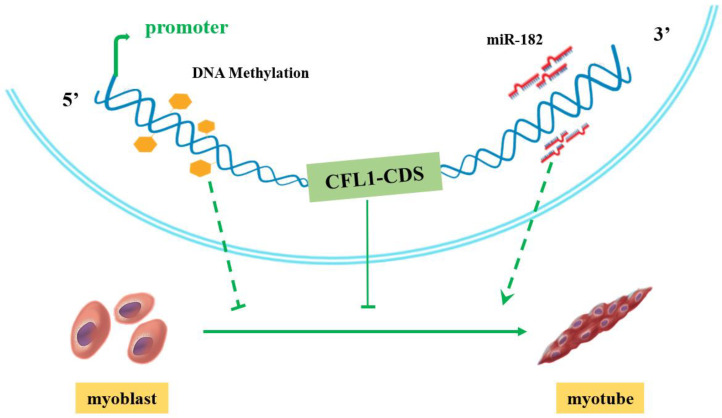
Proposed model of how the *CFL1* gene regulates the differentiation of bovine primary myoblasts.

After transfection with CFL1-CMV, the expression of *CFL1* was gradually upregulated during myoblast differentiation, and the peak values occurred at 5 d after induction (*p* < 0.01). The expression of *MYOD*, *MYOG* and *MYH3* showed a downward trend (Figure 5I). After transfection with shCFL1-2, the expression of *CFL1* slightly decreased during myoblast differentiation. The expression of *MYOD* and *MYOG* was significantly upregulated, and *MYH3* showed a slight increase during myoblast differentiation (*p* < 0.05 or *p* < 0.01). The peak values of *MYOG* and *MYH3* both occurred at 3 d after induction, and *MYOD* occurred at 5 d after induction (Figure 5J). Our experiment comprehensively analyzes the epigenetic regulation mechanisms of *CFL1* gene in the development and differentiation of bovine primary myoblasts (Figure 6).

## 4. Discussion

Muscle is an important factor that affects animal growth and development, and it is one of the crucial indicators to measure meat quality. For cattle, meat quality is an important quantitative characteristic that is regulated by the expression of a series of related genes, and its phenotypes have special regulatory mechanisms and a complex genetic basis. Regulation of gene expression can occur at the level of transcription or translation, affecting the mRNA level and the protein level [20]. Bovine primary myoblasts serve as an excellent model system for studying muscle cell differentiation in vitro. The differentiation of myoblast cells into myocytes or myotubes can be stimulated by changing the serum supplements. Therefore, it is particularly important to further study the molecular genetic regulation network of these genes that regulate muscle growth and development from the perspective of genetics and epigenetics.

Analysis of *CFL1* gene cDNA sequences indicated that *CFL1* has a wide tissue expression spectrum. This observation implies that the *CFL1* gene may have various biological functions in different tissues of the organism. In the same tissues, the expression trend of the *CFL1* gene was revealed at different developmental stages, and the effect of *CFL1* gene expression may vary at three developmental stages. Our results showed that the *CFL1* gene has abnormally high expression in spleen tissues during the calf stage. The spleen is the largest immune organ, and the data indicated that the *CFL1* gene may be involved in mammalian cellular immunity and affect the development of tumors. This result is consistent with a previous report that the *CFL1* gene can participate in cancer development [21,22]. However, the expression level of the *CFL1* gene in adipose tissues was almost unchanged between calf and adult cattle, and the results suggested that the *CFL1* gene was not involved in the regulatory mechanism of growth and differentiation during cattle adipose tissue development. In muscle tissues, the *CFL1* gene showed a low expression pattern in the three developmental stages, and the expression level showed a significant decreasing trend with muscle growth and development. The results further confirmed that the expression of cofilin can gradually transition from the initial non-muscle *CFL1* to muscle *CFL2* with the growth and development of muscle cells [4,23]. However, our data showed that the expression level of the *CFL1* gene was reduced approximately 10 times in adult cattle compared with fetal bovine cattle, and the *CFL1* gene did not completely disappear in the muscle tissues of adult cattle. This result indicated that the *CFL1* gene also plays an indispensable role in muscle growth and development, and the expression change was likely related to the regulatory role of external epigenetics.

Usually, DNA methylation that occurs in the gene promoter region can participate in establishing patterns of gene repression during muscle development and play an important role in the regulation of gene expression. The DNA methylation and expression in adipose and muscle tissues were examined in the differentially methylated region (DMR) of *SERPINA3*. The data showed that DNA methylation patterns had a significant influence on mRNA levels [24,25], and a study has shown that some methylated genes were identified as candidate biomarkers for beef tenderness [26]. In our previous research, *IGF2* expression levels were negatively associated with the methylation status of *IGF2* DMR in heart, spleen, lung and muscle tissues during fetal bovine and adult cattle stages [27]. In this experiment, the expression level of the *CFL1* gene in the AC group was significantly lower than that in the FB group, and the AC group exhibited a significantly higher DNA methylation level than the FB group. There is a noticeable trend for high DNA methylation to reduce gene expression, and more data are required to reveal the exact influence of this tendency. In fetal bovine and adult cattle, the extent of DNA methylation and the expression level of complementary genes in the whole genome and the status of other epigenetic modifications are still unknown. Therefore, additional studies are needed to elucidate the significance of these unknown epigenetic variations in muscle growth and development.

The MiRNAs most likely function independently of other epigenetic mechanisms, and the regulatory mechanisms of epigenetics are mainly combined with specific seed sequences of target genes [28,29]. Studies have shown that miRNAs can be involved in the proliferation and differentiation of skeletal muscle by regulating approximately 1/3 of the genes in the human genome [30]. Due to the numerous miR-182 target genes, the mechanisms of miR-182 involved in the differentiation of skeletal muscle are quite complex and even inconsistent. Some studies have demonstrated that miR-182 may act on the proliferation, migration, and invasion of many cancers through suppression of *FOXO1* [31,32,33]. Our previous experimental results showed that the *FOXO1* gene plays an important role in the differentiation of bovine myoblasts [20]. Recent works have shown that cofilin-1 is a direct target of miR-182-5p in human bladder cancer, and loss of miR-182-5p in bladder cancer can promote cofilin-1 expression [34]. Our research showed that miR-182 can negatively regulate the *CFL1* gene and confirmed the binding site between *CFL1* and miR-182 by dual luciferase assay. After, we found that miR-182 may promote the differentiation of bovine primary myoblasts. This finding provided a basis for studying the function of miR-182 and further exploring whether miR-182 is involved in myoblast differentiation by regulating the role of *CFL1*.

The structure of muscle and the histological characteristics of muscle fibers are closely related to the quality of meat products, and the differentiation of muscle cells plays an important role in promoting the formation of muscle fibers and the development of muscle. In our experimental studies, we confirmed the positive regulatory role of miR-182 in myoblast differentiation, and we preliminarily determined the expression of the *CFL1* gene in myoblast differentiation through the study of the *CFL1* gene and related marker genes in C2C12 cells at different differentiation days.

Muscle differentiation is a multistage process involving cell cycle withdrawal, expression, activation of muscle-specific genes and proteins, and the fusion of mononucleated myocytes into multinucleated myofibers [35]. The peak values of *MYOD*, *MYOG* and *MYH3* appeared at different time points, but then the expression levels all decreased after DM6. Therefore, we can deduce that *MYOD*, *MYOG* and *MYH3* were activated in different stages after myoblast differentiation. There are no reported significant effects of the MYH3 gene after induction in primary bovine skeletal muscle cells. Many published studies have reported that *CFL1* attenuates differentiation in the C2C12 cell line [36,37]. In our study, *CFL1* was gradually downregulated during the differentiation of C2C12 cells, with a slight increase in DM2, when myotubes were completely formed, indicating that *CFL1* tends to suppress the differentiation of myocytes. After, the overexpression and interference vector of the *CFL1* gene was transfected into bovine primary myoblasts to further determine the specific regulatory mechanism of *CFL1* in myoblast differentiation.

Previous studies have shown that the *cofilin* gene can regulate the function and regeneration of normal muscle. Therefore, as a subtype of *cofilin*, non-muscle *CFL1* plays a particularly important role in muscle growth and development. Studies have shown that the expression of *cofilin* changes with the growth and development of healthy mouse muscle cells. In embryonic skeletal muscle, the *CFL1* and *CFL2* genes can be expressed simultaneously. However, with the growth and development of muscle, the CFL1 gene is gradually replaced by the *CFL2* gene at the end of myocyte differentiation [4,9]. The *MYOD, MYOG* and *MYH3* are members of the myogenic regulatory factor MRF family, which can activate muscle gene transcription and drive the transformation of non-muscular cells into muscle cells. The MRFs regulate the occurrence and development of whole muscle, play an important role in myoblast differentiation, and can be used as marker genes in the process of myoblast differentiation to reflect the degree of myoblast differentiation. Our research has showed that the overexpression of the *CFL1* gene had no significant effect on the expression of *MYOD, MYOG* and *MYH3*, indicating that increasing the expression of the *CFL1* gene did not play a significant role in the differentiation of bovine primary myoblasts. However, knocking down the expression of the *CFL1* gene increased the expression of *MYOD, MYOG* and *MYH3* during the differentiation of bovine primary myoblasts, revealing that *CFL1* could suppress myoblast differentiation. This fact may be related to the gradual disappearance of *CFL1* at the end of muscle fiber differentiation. *CFL1* gain-of-function is associated with impaired myoblast differentiation, but loss-of-function can partly restore the inhibition of differentiation by wortmannin [38].

## 5. Conclusions

Our research showed that *CFL1* could suppress the differentiation of bovine primary myoblasts and illustrated the epigenetic regulation mechanisms of the *CFL1* gene in the development and differentiation of bovine primary myoblasts. This study has far-reaching significance to improve the meat production and meat quality of Qinchuan cattle. It can provide reliable data to support and a theoretical research basis for the rapid and efficient breeding selection of local yellow cattle and the genetic improvement of meat quality.

## Figures and Tables

**Table 1:** Primer information for vector construction.

Name	Primer (Contains Protective Bases and Restriction Sites) (5′–3′)
CFL1-CMV-F	CGGggtaccATGGCCTCCGGTGTGGCTGTCT
CFL1-CMV-R	CCCaagcttTCAcatcatcaccatcaccatCAAAGGCTTGCCCTCCAG
shCFL1-1F	gatccCCTCTATGATGCAACCTACTTCAAGAGAGTAGGTTGCATCATAGAGGtttttta
shCFL1-1R	agcttaaaaaaCCTCTATGATGCAACCTACTCTCTTGAAGTAGGTTGCATCATAGAGGg
shCFL1-2F	gatccGGATCAAGCATGAATTACAAGCAAATTCAAGAGATTTGCTTGTAATTCATGCTTGATCCtttttta
shCFL1-2R	agcttaaaaaaGGATCAAGCATGAATTACAAGCAAATCTCTTGAATTTGCTTGTAATTCATGCTTGATCCg
shRNA-NC-F	gatccTTCTCCGAACGTGTCACGTTTCAAGAGAACGTGACACGTTCGGAGAAtttttta
shRNA-NC-R	agcttaaaaaaTTCTCCGAACGTGTCACGTTCTCTTGAAACGTGACACGTTCGGAGAAg

**Table 2:** Primer information for dual luciferase report assay.

Name	Primer (Wild Site Contains Protective Bases and Restriction Sites) (5′–3′)
CFL1-wild-F	CCGCTCGAGACTGCTACGAGGAGGTCAAG
CFL1-wild-R	ATAAGAATGCGGCCGCTCAACCCAAGAGGAATCAAG
CFL1-mut-F	TCCCTTCACCCCAGCAGCCCCCCCGACCC
CFL1-mut-R	TCGGGGGGGCTGCTGGGGTGAAGGGACTG

**Table 3:** Primer information for qRT-PCR.

Name	Primer (Wild Site Contains Protective Bases and Restriction Sites) (5′–3′)
CFL1-F	GTGTGGCTGTCTCTGATG
CFL1-R	CGCTTCTTCACTTCCTCTG
bta-miR-182-RT	GTCGTATCCAGTGCAGGGTCCGAGGTATTCGCACTGGATACGACAGTGTGAG
bta-miR-182-F	ACACTCCAGCTGGGTTTGGCAATGGTAGAA
miR-R	GCAGGGTCCGAGGTATTC
U6-F	GCTTCGGCAGCACATATACTAAAAT
U6-R	CGCTTCACGAATTTGCGTGTCAT
GAPDH-F	AGATAGCCGTAACTTCTGTGC
GAPDH-R	ACGATGTCCACTTTGCCAG

**Table 4:** Primer information for BSP.

Name	Primer (5′–3′)
CFL1-DMR-F	TGTTTTAATAAGGATATTTAGGGTATTT
CFL1-DMR-R	CTCAATAAAAAACTACAACTCAACC

## Data Availability

Not applicable.

## Supplementary Materials

The following supporting information can be downloaded at https://www.mdpi.com/article/10.3390/genes13050723/s1. Figure S1: Agarose gel of electrophoresis pattern of total RNA; Figure S2: Bovine primary myoblasts (×100).
