# Supplementary material for: Epigenetic Regulation Mechanisms of the Cofilin-1 Gene in the Development and Differentiation of Bovine Primary Myoblasts
Source: Genes (Basel). 2022 Apr 21;13(5):723. doi: 10.3390/genes13050723 (PMC9140398; doi:10.3390/genes13050723)
Supplement: Supplementary file 1 [file genes-13-00723-s001.zip › genes-1674991-supplementary.pdf]

[redacted]

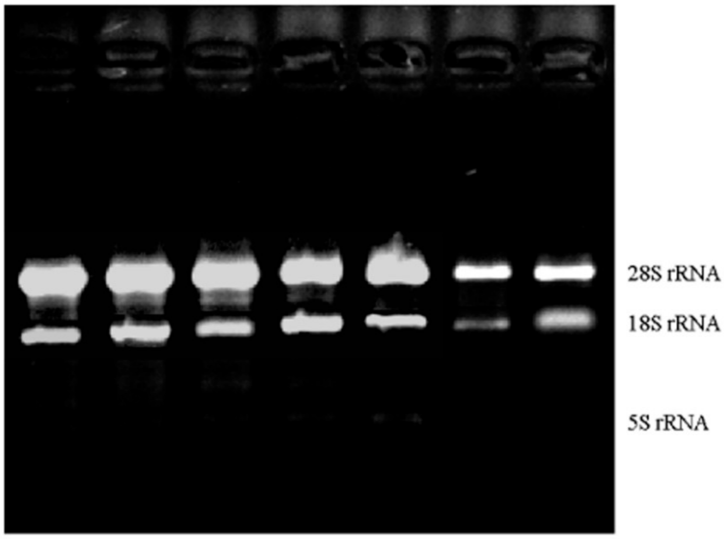

Figure S1. Agarose gel of electrophoresis pattern of total RNA.

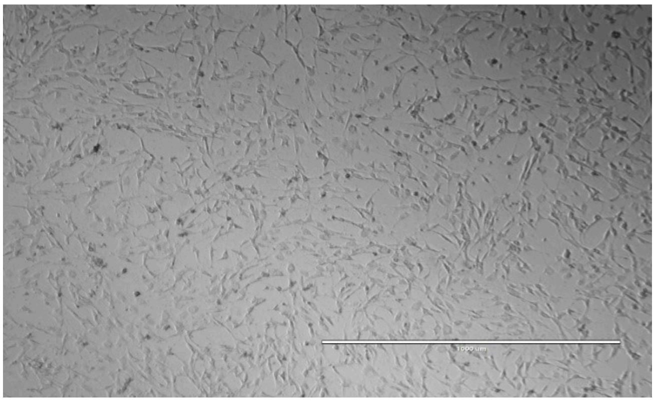

Figure S2. Bovine primary myoblasts (×100).
